# Simulation-Comparative Approach to Description of Glass Structural Relaxation Dynamics

**DOI:** 10.1021/acsomega.6c05942

**Published:** 2026-07-30

**Authors:** Roman Svoboda

**Affiliations:** Faculty of Chemical Technology, Department of Physical Chemistry, 48252 University of Pardubice, Studentská 573 532 10, Pardubice, Czech Republic

## Abstract

Accurate description of structural relaxation processes is essential for predicting long-term stability of chalcogenide glasses utilized in memory, photonic, and infrared-optical applications. Although the Tool–Narayanaswamy–Moynihan (TNM) model remains one of the most effective frameworks for describing the glass transition kinetics, determination of its nonlinearity (*x*) and nonexponentiality (β) related features is often hindered by instrumental distortions and instability of the conventional curve-fitting procedures. Here, a robust and user-friendly implementation of the simulation-comparative method for extraction of the *x* and β TNM parameters is presented. An extensive library of precomputed core data sets is introduced, covering physically relevant ranges of enthalpy relaxation activation energy (200–500 kJ·mol^–1^) and glass transition temperatures (−50–450 °C) for chalcogenide glasses. Utilization of these data sets in terms of the presented simulation-comparative method enables rapid, fitting-free estimation of *x* and β by direct comparison with experimental calorimetric data, using only basic data-processing tools. The predictive performance of the method was systematically validated—using defined and randomly generated theoretically simulated data sets, as well as experimental calorimetric data extracted from the literature—the method predicts the parameters *x* and β with an uncertainty of ±0.05, further decreasing this uncertainty to ±0.02 for the most relevant cases of the relaxation behavior. By eliminating the need for specialized software and unstable optimization routines, the presented approach substantially lowers the barrier for routine TNM analysis and enables reliable monitoring of subtle structural relaxation trends in chalcogenide glasses. The provided core data sets, together with accompanying MATLAB code, establish a practical and broadly applicable platform for structural relaxation studies in amorphous chalcogenide materials.

## Graphical abstract

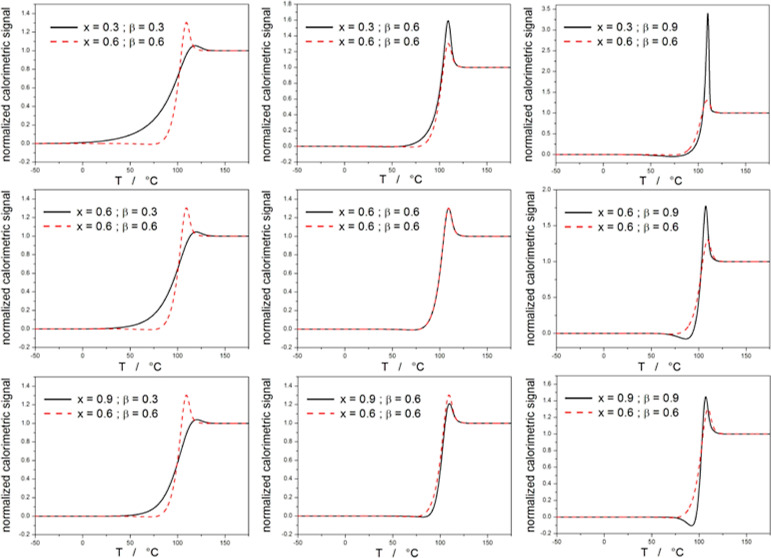

## 1 Introduction

Amorphous chalcogenide materials, composed primarily of chalcogen elements (S, Se, Te) combined with network formers (e.g., As, Ge, Sb, Ga), are widely used due to their unique optical and electronic properties.^1–3^ One of their most important applications is in phase-change memory and data storage technologies, where their ability to reversibly switch between amorphous and crystalline states enables fast, nonvolatile information storage.^4–8^ They are also extensively used in infrared optics, including lenses, fibers, and windows, because of their high transparency in the mid- to far-infrared region.^9–11^ In addition, amorphous chalcogenides play a key role in photonics and optoelectronics, such as optical waveguides, all-optical switches, and nonlinear optical devices, owing to their high refractive indices and strong nonlinear responses.^12,13^ Other applications include thin-film solar cells,^14,15^ photoreceptors in xerography,^16^ and chemical or biological sensors, where their tunable electrical conductivity and sensitivity to light and environmental changes are particularly advantageous.^17–19^ The common feature of all these applications is the necessity of long-term stability of the glassy/amorphous state, which is dictated by the structural relaxation phenomena. Hence the importance of the ability to predict the trends/drifts in structural arrangements associated with the relaxation movements below the glass transition temperature (*T*_g_), which can be only achieved by accurate modeling of the glass transition kinetics.

1 $$\Phi \left(t\right)=\exp\left[-{\left(\int_{0}^{t}\frac{dt}{\tau \left(T,T_{f}\right)}\right)}^{\beta }\right]$$

The Tool–Narayanaswamy–Moynihan (TNM) model^20–22^ is nowadays, despite its shortcomings,^23–25^ still one of the most commonly used and best-performing concepts for the description of structural relaxation behavior in the glass transition range.^26–34^ The TNM model is expressed by the following set of equations

2 $$\tau \left(T,T_{f}\right)=A\cdot \exp\left[x\frac{\Delta h^{*}}{RT}+\left(1-x\right)\frac{\Delta h^{*}}{RT_{f}}\right]$$

where Φ­(*t*) is the relaxation function of the given property, *t* is the time, τ is the relaxation time, β is the nonexponentiality parameter, *A* is the pre-exponential factor, *x* is the nonlinearity parameter, Δ*h* ^*^ is the apparent activation energy of the structural relaxation, *R* is the universal gas constant, *T* is the temperature, and *T*_*f*_ is the fictive temperature, which is defined as the temperature of the undercooled liquid with the same structure as that of the relaxing glass. Note that the present paper will primarily consider the derivative data, i.e., dT_f_·dT^–1^, that are accessible by the most common relevant instrumental technique—differential scanning calorimetry (DSC). In such a depiction, the Δ*h* ^*^ and A parameters are primarily responsible for the position and width of the relaxation peak with respect to the T axis, and the parameters x and β determine the height of the relaxation peak (see Figure 1 for a visual example).

**Figure 1:**
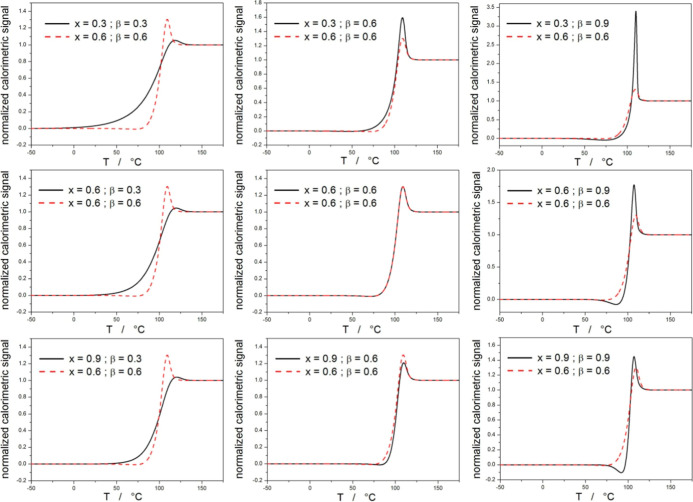
Example of the impact of TNM parameters *x* and β on the dT_f_/dT signals conformant to the normalized calorimetric data measured during the heating scans (at 10 °C·min^–1^) following the cooling from 200 to 0 °C at 10 °C·min^–1^. The other two TNM parameters used in the simulations were Δ*h* ^*^ = 300 kJ·mol^–1^ and ln­(A/s) = −93. The data curve for *x* = 0.6 and β = 0.6 (red dashed curve) serves as an identical comparative benchmark.

As a phenomenological model, the TNM concept is highly flexible and capable of accurately accounting for the main features of the glass transition behavior—the hysteresis, nonlinearity, and nonexponentiality.^35,36^ The phenomenological nature of the TNM model can be perceived as a certain disadvantage, but in the past decade, the interpretation of the TNM parameters (Δ*h* ^*^, A, *x*, β) was largely improved,^37–41^ to the point of the particular structural movements and their interactions being associated with certain TNM parameters. As the structural relaxation processes can manifest through diverse measurable properties, including thermal/enthalpic signatures accessible by calorimetric protocols and analyses,^37–43^ relaxation-linked density/volume (mass–thickness) evolution in thin-film geometries,^44^ and optical/optoelectronic changes in functional amorphous films and molecular glasses,^45,46^ the corresponding instrumental and methodological advancements (e.g., broadband dielectric spectroscopy, quasi-elastic scattering, or broadened structural-analysis tool sets such as pair distribution function analysis) enable structural relaxation measurements and characterization across an increasingly broad range of materials,^47,48^ spanning polymers and major oxide glass families (e.g., silicate, phosphate, or borate glasses),^32,40,49^ as well as niche systems such as amorphous pharmaceutical glass formers,^37,42,50^ organic/molecular glasses and semiconducting thin films for optoelectronics,^44,46,51^ and ultrathin functional chalcogenide film systems.^39,45,52^ With the structural relaxation studies being more and more readily included in the standard materials research, an increased demand for methods enabling precise determination of the TNM model parameters occurs, which would allow monitoring of subtle relaxation behavior changes with compositional tuning.

In 2013, a comprehensive guide to the determination of the TNM model parameters (Δ*h* ^*^, A, *x*, β) was published.^53^ Although it suggests the curve-fitting approach (nonlinear optimization utilizing the least-squares criterion) as the dominant way of extracting the TNM parameters from the experimental structural relaxation data, this approach can often be unreliable/unusable due to the intrinsic data-distortive effects originating either from instrumental artifacts, experimental conditions, or material relaxation characteristics.^23,25,53–55^ It is namely the values of *x* and β, which are in such cases burdened by systematic errors, with the optimization procedure often failing, providing physically unjustified values of these parameters (due to their divergence toward arbitrary boundaries—usually 0 or 1). Therefore, the alternative nonfitting methods (as described in ref 53) are of great importance in the field of structural relaxation (glass transition) kinetics. Among these methods, the simulation-comparative approach^56^ and its extended version^57^ are the most potent and robust ways of reliably and simultaneously determining *x* and β TNM parameters.

Both these simulation-comparative methods are based on either numerical^56^ or visual^57^ comparison of the heights of the relaxation peaks/overshoots (*C*_p_^max^) for the experimentally measured and theoretically simulated data. Whereas the calorimetric measurement of the experimental enthalpy relaxation data is straightforward and relatively simple to perform, the simulation of the corresponding data set of the theoretical *C*_p_^max^ values is not trivial. First, the TNM model (eqs 1 + 2) needs to be simulated accounting for the thermal history—the TNM integration is performed by explicitly summing the entire past temperature history at each time step, using a backward numerical integration of reduced time ∫dt/τ and a stretched-exponential kernel to weight each temperature increment’s contribution to the fictive temperature.^58^ Second, an extensive amount of data needs to be simulated for the simulation-comparative method to perform with acceptable precision. These two aspects appear to be the major hindrances for the widespread accommodation of the structural relaxation description in nowadays studies dealing with amorphous material research.

In the present paper, an extensive collection of theoretically simulated data will be introduced, using which the simulation-comparative method can be used very quickly, efficiently, and to a great degree of accuracy, without the need for utilization of any specialized software (other than a common table editor such as MS Excel). In addition to the detailed description of the data and associated computations estimating the *x* and β values (so that they are readily accessible to a nonexpert in the field), the predictive accuracy of the present simulation-comparative methodology will be thoroughly tested and reported, and the corresponding MATLAB code for the TNM model simulation will be provided. The ranges for the simulated data matrix will respect the physically meaningful boundaries characteristic of the chalcogenide materials (with the corresponding justification being provided). Apart from the extensive theoretical testing, the introduced procedure will also be verified by applying it to the selected experimental data sets collected by the author and his associates over the past decades.

## 2 Simulation-Comparative Method + Core Data Sets

As mentioned in the introductory part, the simulation-comparative method is based on the comparison of the experimentally measured results with an extensive matrix of theoretically simulated data, where the best match indicates the correct set of *x* and β values. The utilized temperature program consists of the constant heating rate (CHR) cycles,^55^ where the sample is periodically cooled from the undercooled liquid state (above *T*_g_) to the glassy state (well below *T*_g_) and then immediately heated back above *T*_g_. During these alternating cooling and heating steps, the cooling rates (q^–^) vary (so that always a different T_f_ is reached during cooling), while the consequent heating rates stay constant (usually 10 °C·min^–1^, which is a good compromise between the signal strength, time-demandingness, and thermal gradient occurrence). Schematically, the CHR cyclic temperature program is shown in Figure 2A, and the corresponding derivative kinetic data (conformable to, e.g., calorimetric measurements) are shown in Figure 2B. In practice, lower cooling rates are preferred due to significantly lower T_f_s being reached, which in turn results in higher and more pronounced relaxation peaks/overshoots during the consequent heating step. The raw calorimetric data (such as obtained e.g. by differential scanning calorimetry DSC or differential thermal analysis DTA) need to be normalized first to remove the influence of the absolute values of heat capacity c_p_. The normalized heat capacity C_p_^N^ is calculated as

3 $$C_{p}^{N}=\frac{dT_{f}}{dT}=\frac{C_{p}-C_{pg}}{C_{pl}-C_{pg}}$$

where *c*_p_(*T*) is the measured calorimetric/heat capacity signal, and *c*_pg_(*T*) and *c*_pl_(*T*) are the extrapolated heat capacity signals stabilized in the glassy (below *T*_g_) and undercooled liquid (above *T*_g_) regions. It is worth noting that the *c*_pg_(*T*) and *c*_pl_(*T*) dependencies do not necessarily have to be linear, and their extrapolation can be done by using polynomial functions.

**Figure 2:**
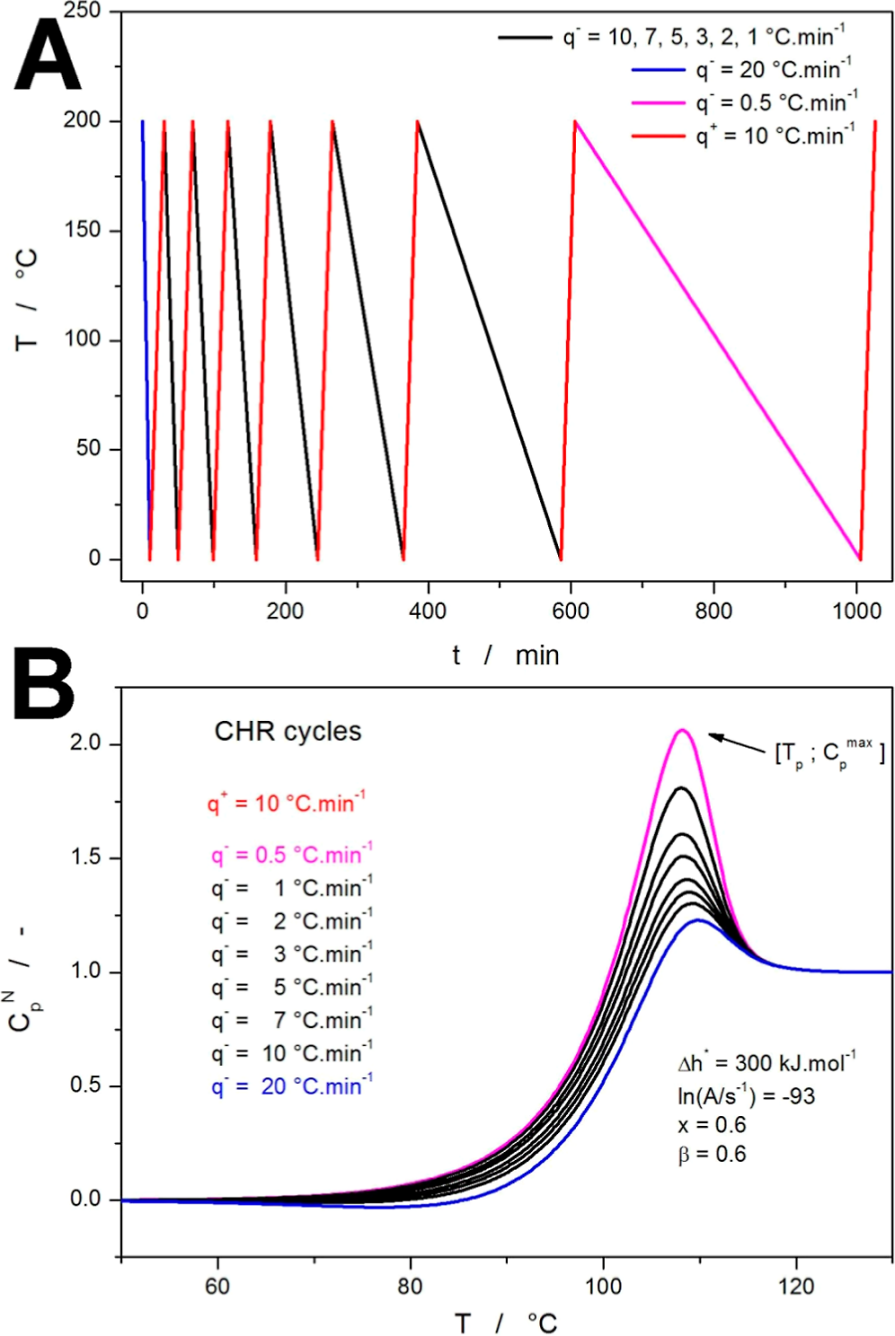
(A) Example of the CHR cyclic temperature program employing a variety of cooling rates and one heating rate. (B) *C*_p_^N^-T dependencies obtained during the heating scans of the CHR cycles depicted in graph A; the TNM parameters used for the simulations, and the identification of the *T*_p_ and *C*_p_^max^ coordinates evaluated for the curve with the largest relaxation peak are indicated in the graph.

To perform the comparison with the experimental data (set of CHR heating scans normalized to *C*_p_^N^-T dependencies), their potential theoretical counterpart had to be simulated. The corresponding algorithm for simulating data in terms of the TNM model was taken from ref 58, with eqs 4 and 5 representing nonisothermal and isothermal steps in the temperature program, respectively

4 $$T_{f,n}=T_{0}+\sum_{j=1}^{n}\Delta T_{j}\left\{1-\exp\left[-{\left(\sum_{k=j}^{n}\Delta T_{k}/q_{k}\tau_{k}\right)}^{\beta }\right]\right\}$$

5 $$T_{f,n}=T_{0}+\sum_{j=1}^{n_{A}}\Delta T_{j}\left\{1-\exp\left[-{\left(\sum_{k=n_{A}}^{n}\Delta t_{e,k}/\tau_{k}\right)}^{\beta }\right]\right\}$$

where *T*_0_ is the initial equilibrium temperature above *T*_g_, *t*_*e*_ is the annealing time, and τ is the relaxation time calculated according to eq 2. The Boltzmann superposition integral over time (replaceable by the corresponding integral over temperature) is used to determine *T*_*f*_. The continuous cooling or heating is thus replaced by a sequence of *n* temperature jumps Δ*T*, followed by isothermal holds with duration determined by the cooling and heating rates Δ*t* = Δ*T*/*q*. The magnitude of Δ*T* = 0.2 °C was used for the present simulations to ensure sufficient accuracy of the simulated data. The self-retarding kinetics was introduced by dividing the aging time into *k* subintervals and calculating *T*_f_ and τ at the end of each. The *C*_p_^N^ quantity was then obtained by simple derivation of *T*_f_ (see eq 3). The corresponding code for MATLAB simulation is included as the Supporting Information.

The concept of the standard simulation-comparative method is based on the experiment/vs/simulation comparison of the heights of the relaxation peaks (*C*_p_^max^; see Figure 2B for visual explanation) for a series of CHR cycles. Since the commonly applied q^–^ during the CHR cycles range between 0.5 and 20 °C·min^–1^, the present simulated data cover an identical range—namely, 0.5, 1, 2, 3, 5, 7, 10, and 20 °C·min^–1^. Note that the user does not have to perform the full set of the eight CHR cycles defined by the above-mentioned q^–^ values; any number and combination of q^–^ applied during the CHR cycles is suitable for the utilization of the present data sets. The heating rate q^+^ used for the simulations was 10 °C·min^–1^, conformable to the standard choice of the majority of the DSC users. To cover the full span of possible *x* and β combinations, the matrix was explored in the 0.1–0.98 range with a step of 0.02 for each of the two TNM parameters, resulting in 2025 different *x* and β combinations. For each of these 2025 combinations, the set of 8 CHR cycles was simulated and the corresponding *C*_p_^max^ values were extracted.

In addition to the above-mentioned parameters, the simulations of the C_p_^N^-T dependencies require the knowledge of the independently determined Δ*h* ^*^ and A values and the implementation of the identical temperature program as was used for the experiment (choice of the correct temperature interval and cooling and heating rates). Ideally, this should be done for the exact values characterizing the given material/experiment, i.e., the true Δ*h* ^*^ and A values and the exact *T*_max_ and *T*_min_ temperatures characterizing the temperature range of the experiment. This is, however, of course feasible only with the proper specialized software and deeper understanding of the relaxation phenomena, which is not accessible to the vast majority of researchers dealing with glassy/amorphous materials. The idea of the present paper is to bypass these requirements by simulating only a limited number of the large/core data sets (each containing the *C*_p_^max^ data for the 2025 sets of *x* and β combinations) for a sufficient number of Δ*h* ^*^ and A combinations and showing that using the data set simulated for the closest Δ*h* ^*^ and A values results in acceptably accurate estimates of *x* and β parameters. Note that the values of Δ*h* ^*^ and A have only very little effect on the *C*_p_^max^ values (which are the key quantity employed in the simulation-comparative method). In practice, the user will only need to determine/estimate the activation energy Δ*h* ^*^ for the studied material (which can be easily done by simple linearization method described in ref 54—details will be given in the short TLDR guide located in the concluding section) because each of the core data sets is characterized by the Δ*h* ^*^ and *T*_p_ values, where *T*_p_ is the temperature corresponding to the maximum of the relaxation peak. Note that the pre-exponential factor A is (together with Δ*h* ^*^) responsible for the localization of the glass transition effect on the *T* axis and can thus be effectively replaced (for matching purposes) by any convenient temperature characteristics (*T*_p_ in the present case). Hence, the user can choose the closest theoretically simulated data set matching his/her experimental data only based on the Δ*h* ^*^ and *T*_p_ values, which both are very well accessible and easily determined. All simulations were automatically performed in broad-enough temperature ranges so that the user does not have to be concerned with considering the match between the experimental *T*_max_ and *T*_min_ values and those used for the simulations.

The exact choice of the Δ*h* ^*^ and A combinations used to simulate the present core data sets was based on the typical ranges of these two quantities for the glassy/amorphous chalcogenides. Regarding Δ*h* ^*^, it was shown in the recent review^32^ (dealing with the chalcogenide materials characterized in terms of the TNM model) that for the ∼80 listed compositions, Δ*h* ^*^ ranges between 200 and 500 kJ·mol^–1^. An identical range can also be derived from the review^59^ reporting on the viscosity of the chalcogenide glass formers, where the activation energy of structural relaxation Δ*h* ^*^ can be estimated based on the assumed equality^60,61^ with the activation energy of viscous flow *E*_η_ determined in the glass transition range. Here, the only exceptions are the lower value of ∼150 kJ·mol^–1^ reported for certain As–S glasses and the higher value of ∼600 kJ·mol^–1^ reported for certain (Ga_2_S_3_)-(La_2_O_3_) glasses. Nonetheless, even these outliers can be safely included in the present version of the simulation-comparative evaluations, as will be shown in Section 3. The span of the pre-exponential factor values was then set based on the corresponding *T*_g_ values (as described above), where the lowest reported *T*_g_s are for the pure sulfur and S-rich chalcogenides (*T*_g_s ≈ −30 °C), and the highest reported *T*_g_s are for the GeS_2_-based chalcogenides (*T*_g_s up to 480 °C).^32,59^ These Δ*h* ^*^ and *T*_g_(*T*_p_) ranges were thus reflected in the choice of suitable combinations Δ*h* ^*^ and A covering the whole matrix of options relevant for the chalcogenide glasses. As a result, 56 core data sets (7 Δ*h* ^*^ values × 8 temperatures) were simulated as the fundamental basis for the application of the simulation-comparative method—the characteristics of these data sets are listed in Table 1.

**Table 1:** Characterization of Core Data Sets Simulated as a Basis for the Simulation-Comparative Evaluations^a^

Δh*/kJ·mol^–1^	200	200	200	200	200	200	200	200
*T*_p_/°C	–55	5	70	112	165	230	325	455
ln(A/s)	–110	–86	–69	–61	–53	–46	–38	–30
Δh*/kJ·mol^–1^	250	250	250	250	250	250	250	250
*T*_p_/°C	–55	5	70	112	165	230	325	455
ln(A/s)	–138	–107	–87	–77	–67	–58	–48	–39
Δh*/kJ·mol^–1^	300	300	300	300	300	300	300	300
*T*_p_/°C	–55	5	70	112	165	230	325	455
ln(A/s)	–165	–129	–104	–93	–81	–70	–58	–47
Δh*/kJ·mol^–1^	350	350	350	350	350	350	350	350
*T*_p_/°C	–55	5	70	112	165	230	325	455
ln(A/s)	–193	–151	–122	–109	–95	–82	–69	–56
Δh*/kJ·mol^–1^	400	400	400	400	400	400	400	400
*T*_p_/°C	–55	5	70	112	165	230	325	455
ln(A/s)	–220	–173	–139	–124	–109	–94	–79	–64
Δh*/kJ·mol^–1^	450	450	450	450	450	450	450	450
*T*_p_/°C	–55	5	70	112	165	230	325	455
ln(A/s)	–248	–195	–158	–140	–123	–106	–89	–72
Δh*/kJ·mol^–1^	500	500	500	500	500	500	500	500
*T*_p_/°C	–55	5	70	112	165	230	325	455
ln(A/s)	–270	–216	–175	–156	–137	–118	–99	–81

a Each data set is characterized by the Δh* + *T*_p_ + ln a triplet. The listed *T*_p_ values are the intended entries; due to the rounding of the ln­(A/s) values, which were truly used for the simulations, the true *T*_p_s may differ by a few °C from the intended values. The true *T*_p_ values are included in the names of the core data set files.

Each core data set contains 2025 entries/rows with the following structure: “0.54 0.74 2.870882 2.406884 2.051454 1.886048 1.716378 1.625446 1.544627 1.426498”, where the first two numbers correspond to the *x* and β values, respectively, and the following eight numbers indicate the *C*_p_^max^ values for the CHR cycles characterized by *q* ^–^ = 0.5, 1, 2, 3, 5, 7, 10, and 20 °C·min^–1^, respectively (with *q* ^+^ = 10 °C·min^–1^). All core data sets are included as TXT files (using space separator) in the Supporting Information—the corresponding naming policy is “_250 – 55”, where the first number after the underline denotes Δh* in kJ·mol^–1^, and the second number after the space indicates *T*_p_ in °C (−55 °C for the given example).

## 3 Validation of the Simulation-Comparative Method

The present section will be structured into three sub-sections: (1) validation of the methodology using a defined simulated data set; (2) validation of the methodology using a random simulated data set; and (3) validation of the methodology using real-life experimental data taken from the literature.

### 3.1 Validation Using a Defined Simulated Data Set

The validation of the simulated core data sets was done in a way that replicates the procedure that the ordinary user will adopt—i.e., with known (predetermined) Δ*h* ^*^ and *T*_p_, the core data set with the closest matching values of these two quantities is selected (with the priority given to the Δ*h* ^*^ value, and *T*_p_ being the secondary criterion), and then the least-squares analysis is applied to select the set of *C*_p_^max^ values from the core data set that best matches the experimental data

6 $$SSR=\sum_{i=q_{\min}^{-}}^{q_{\max}^{-}}{\left(C_{p,\mathrm{EXP}}^{\max}-C_{p,CORE}^{\max}\right)}^{2}$$

where the summation for the sum of squared residue (SSR) is performed over all applicable data pairs defined by different *q* ^–^, and the EXP and CORE indices denote the *C*_p_^max^ values obtained experimentally and extracted from the selected core data set, respectively. The *x* and β values that were used to simulate this best matching set are then identified as the most accurate available estimates of these TNM parameters.

To test the accuracy of the simulation-predictive method employing the 56 core data sets described in Section 2, an extensive series of *C*_p_^max^ eights (corresponding to the 8 above-defined *q* ^–^) were prepared and used as the “experimental” data. In particular, since the testing was primarily focused on the inaccuracies associated with the core data sets having sparsely spaced Δ*h* ^*^ and *T*_p_ values, the present testing series were simulated for a large number of Δ*h* ^*^ and *T*_p_ combinations uniformly covering the whole Δ*h* ^*^ and *T*_p_ ranges relevant for the amorphous chalcogenide materials. These 976 combinations are explicitly listed in the Supporting Information; the Δ*h* ^*^ values change between 200 and 500 kJ·mol^–1^ with a step of 20 kJ·mol^–1^ (giving 16 different Δ*h* ^*^ values), and for each Δ*h* ^*^ value, 61 distinct *T*_p_ temperatures were selected, evenly covering the −55 – 465 °C range. For each of these 976 Δ*h* ^*^ and T_p_ combinations, only 9 *x* and β combinations were employed, composed of *x* and β equal to 0.3, 0.6, or 0.9—these values practically cover the most important experimentally encountered ranges, with the most relevant combinations being *x* = 0.3 and β = 0.6 and *x* = 0.6 and β = 0.6. In this way, 8784 testing series of the *C*_p_^max^ eights (corresponding to the CHR cycles with *q* ^–^ = 0.5, 1, 2, 3, 5, 7, 10, and 20 °C·min^–1^) were prepared. For each series, the estimation of *x* and β was performed via the present simulation-comparative method based on the 56 core data sets—the results are shown in Figure 3.

**Figure 3:**
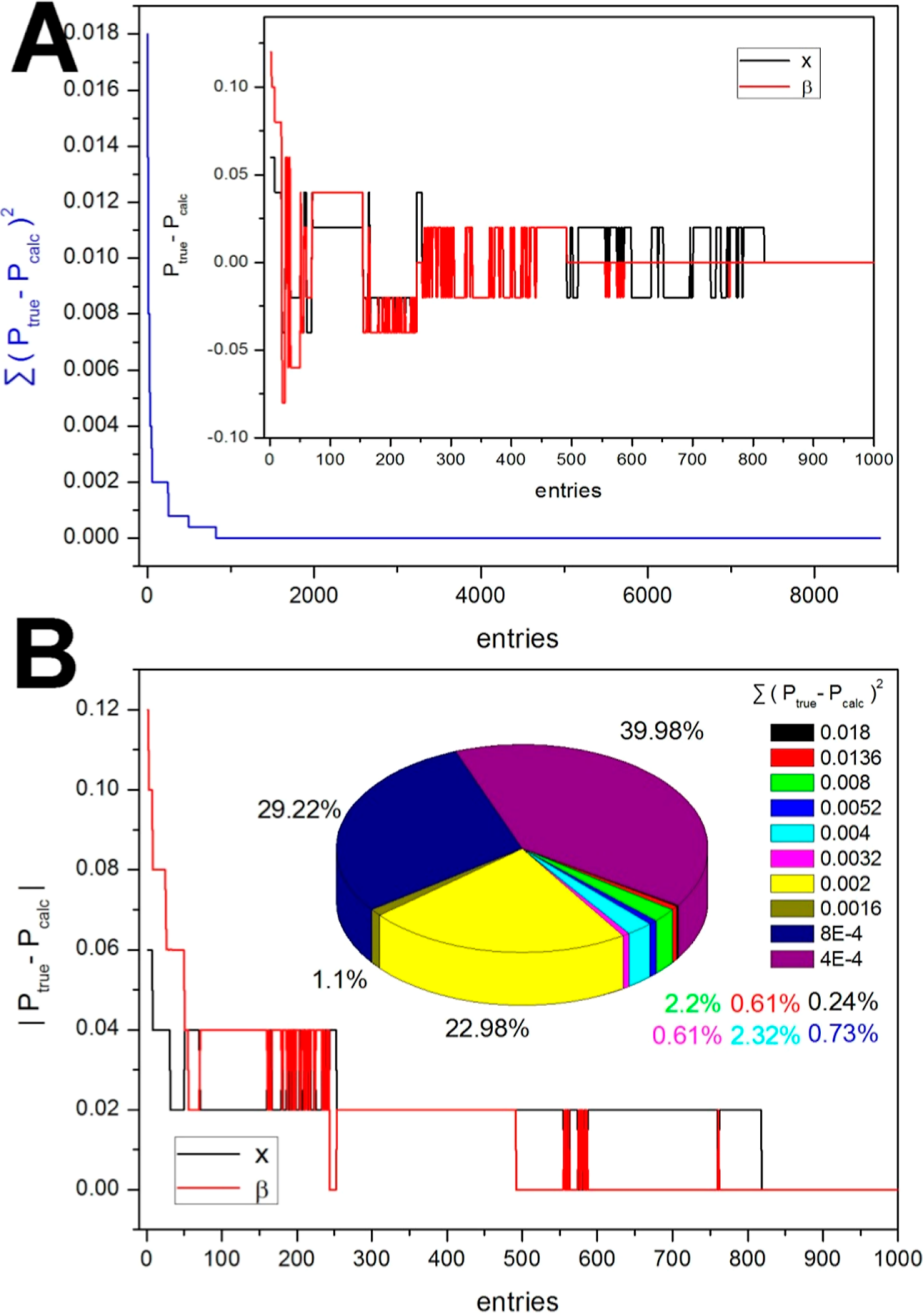
(A) Sum of squared errors obtained within the first validation testing for the considered parameters P (*x* and β); the inset shows the errors for the individual parameters. The errors were sorted from the largest to the smallest. (B) Absolute values of the errors from the inset of graph A. The inset in graph B shows the percent representation of the magnitudes of the sums of squared errors—the percent was calculated only from the entries with nonzero errors.

The main graph in Figure 3A shows the sum of squared residue defined as

7 $${SSR}_{valid}={\left(x_{true}-x_{calc}\right)}^{2}+{\left(\beta_{true}-\beta_{calc}\right)}^{2}$$

where the indices “true” denote the true values of *x* and β (used to simulate the given series out of the 8784 testing ones), and the indices “calc” denote the values of *x* and β estimated via the present simulation-comparative method. The residues are sorted from the largest to the smallest. Note that since the core data sets were prepared with the 0.02 steps in *x* and β, the “calc” estimates will always be multiples of 0.02 when compared against the true values of 0.3, 0.6, and 0.9. The inset in Figure 3A shows the absolute values of the individual *x* and β residues; the graph is zoomed in on the nonzero residues, and due to the sorting process, the randomness of the positive/negative residues was partly lost. The absolute values of the individual residues are shown in Figure 3B, where the inset depicts the representation of the particular SSR_valid_ values within the pool of all nonzero sums of squared residues. The content of the main graph from Figure 3B is listed in Table 2 in percent calculated out of all 8784 testing entries.

**Table 2:** Representation of the Distribution of the Combined Errors/Deviations in the *x* and β Estimation Depicted in Figure 3A

errors	∑(*P*_true_-*P*_calc_)^2^	incidence/%
0.12 + 0.06	0.018	0.023
0.10 + 0.06	0.0136	0.057
0.08 + 0.04	0.008	0.205
0.06 + 0.04	0.0052	0.068
0.06 + 0.02	0.004	0.216
0.04 + 0.04	0.0032	0.057
0.04 + 0.02	0.002	2.140
0.04	0.0016	0.102
0.02 + 0.02	0.0008	2.721
0.02	0.0004	3.723
0	0	90.688

Based on these data, the following conclusions can be derived. All error combinations with ∑(*P*_true_-*P*_calc_)^2^ ≥ 0.0032 were obtained exclusively for β_true_ = 0.9 and *x*_true_ = 0.3 (with few exceptional cases of *x*_true_ = 0.9). Since these combinations describe extremely rare, in real-life materials practically never-encountered types of structural relaxation behavior, it can be stated that the present version of the simulation comparative method should be able to predict the TNM parameters *x* and β with a ±0.04 accuracy, with over 97% of the data being predicted with a ±0.02 accuracy. Within the framework of the present testing range (combinations of *x* and β equal to 0.3, 0.6, and 0.9), the parameter *x* is being predicted more accurately with regard to | *P*_true_-*P*_calc_ |—the highest deviations are of the 0.06 magnitude—but at the same time, the incidence of the lower (0.02) errors is approximately double that for the β parameter.

### 3.2 Validation Using a Randomly Simulated Data Set

The second way of the core data set validation was done via a similar approach as described in Section 3.1, only instead of the precisely defined testing data series (with arbitrarily selected Δ*h* ^*^, A, *x*, and β values), 15,000 sets of randomly selected combinations of the four TNM parameters were used to simulate the testing data series. The Δ*h* ^*^ values were evenly distributed in the 200–500 kJ·mol^–1^ range (5000 sets per 100 kJ·mol^–1^ interval); ranges for the random selection of the A values were set to cover the −50–450 °C range for each of the three Δ*h* ^*^-based intervals; the values of *x* and β were randomly selected from the 0.20–0.95 ranges. Otherwise, exactly same simulation and evaluation procedures as described in the previous section were adopted: (1) for each of the 15,000 combinations of the TNM parameters, the *C*_p_^max^ eights (corresponding to the CHR cycles with *q* ^–^ = 0.5, 1, 2, 3, 5, 7, 10, and 20 °C·min^–1^) were simulated; (2) the closest core data set was selected based on the Δ*h* ^*^ and *T*_p_ values (with Δ*h* ^*^ being prioritized); (3) the best matching set of *C*_p_^max^ values from the selected core data set was attributed to the considered testing data set (one of the 15000) using eq 6; (4) the corresponding *x* and β estimates were statistically processed similarly as described by eq 7 and Figure 3 in Section 3.1. The results of this second validation test are shown in Figure 4.

**Figure 4:**
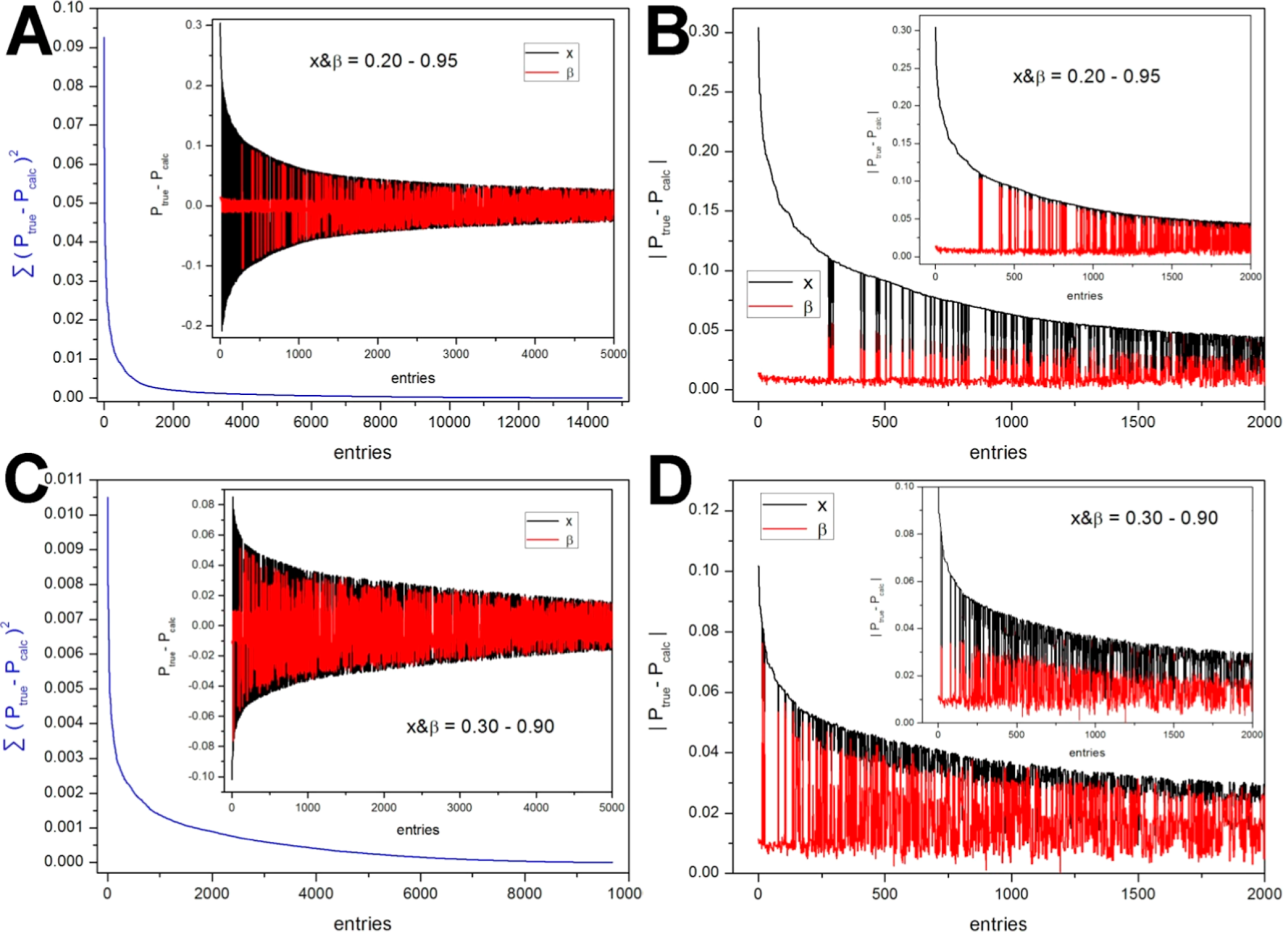
(A) Sum of squared errors obtained within the full second validation testing (with *x* and β ranging between 0.20 and 0.95) for the considered parameters P (*x* and β); the inset shows the errors for the individual parameters. The errors were sorted from the largest to the smallest. (B) Absolute values of the errors from the inset of graph A. The inset in graph B shows an identical graph as the main figure—only the data layer for the β parameter is now put forward. (C) Sum of squared errors obtained within the truncated second validation testing (with *x* and β ranging between 0.30 and 0.90) for the considered parameters P (*x* and β); the inset shows the errors for the individual parameters. The errors were sorted from the largest to the smallest. (D) Absolute values of the errors from the inset of graph C. The inset in graph D shows an identical graph as the main figure—only the data layer for the β parameter is now put forward.

Profile-wise, the distribution of errors shown in Figure 4A is strongly reminiscent of that from Figure 3A. However, in absolute values, the errors from the present test are almost 1 order of magnitude higher, which is caused by the broadening of the testing ranges to 0.20–0.95 (compared to the borderlines being 0.3 and 0.9 in the first testing series reported in Section 3.1). Indeed, the absolute majority of the highest errors are associated with β ≤ 0.26 and *x* ≥ 0.6. As is shown in the zoomed-in graph in Figure 4B, where the inset depicts the identical graph is the main boy of the graph, only the dependencies for *x* and β are switched with respect to their foreground/background position, it is clear that in these high-error cases, it is dominantly the *x* value that gets estimated incorrectly due to the low resolution in *C*_p_^max^ (which is a consequence of the very low β values). Since such low β values are typical only for a very limited number of amorphous chalcogenide materials with a very high degree of cross-linking (e.g., Ge_15_Se_85_ ^57^), for which the *x* values are typically similarly low (and certainly not higher than 0.5, otherwise the calorimetric measurement would not exhibit a clearly recognizable relaxation peak and the simulation-comparative method would not be applicable anyway), these *x* and β combinations are irrelevant for practice anyway.

Hence, Figure 4C,D shows the analysis of the present simulation-comparative method performance limited to the 0.3–0.9 range of the testing *x* and β values (9685 cases), which are relevant from the practical point of view. Here, the errors are already comparable to those shown in Section 3.1 for the first validation test. Out of these 9685 testing *x* and β combinations, more than 97% were estimated with an error below 0.05, and more than 80% with an error below 0.03. As is apparent from Figure 4D, the errors in estimating the parameter *x* are on average higher and more frequent compared to those associated with β estimation. Except for the above-mentioned direct correspondence between the high *x* and β estimation errors and very low β + high *x* combinations, no other significant simple/linear correlations between the magnitude of the estimate errors and input data were revealed; the tested quantities were individual TNM parameters, “β – *x*”, “Δh*—*T*_p_”, “|(β – *x*)·(Δh*—*T*_p_)|“, “Δh*/ |*T*_p_|“, “|Δh*_true_—Δh*_calc_|+|*T*_p,true_ – *T*_p,calc_|“, “β/*x*”, “(β/*x*)·(Δh*—*T*_p_)”. Accordingly, a relatively complex relationship can be assumed for identifying the materials with potentially more inaccurately estimated *x* and β parameters; hence, it needs to be assumed that the precision of the *x* and β estimates via the present version of the simulation-comparative methodology is ±0.05. Nonetheless, it is important that the “|Δh*_true_—Δh*_calc_|+|*T*_p,true_ – *T*_p,calc_|” quantity has very low correlation with the magnitude of the estimate errors, which indicates that the present resolution of the core data sets with respect to Δh* and A (or *T*_p_) is sufficient.

### 3.3 Validation by Comparison with Experimental Data from the Literature

To validate the present simulation-comparative methodology also using data for real-life materials, the TNM parameters reported for the chalcogenide glasses in ref 32 were taken together with the *C*_p_^max^-*q* ^–^ data extracted from the references listed therein. Note that only those materials for which the CHR cycles were measured with *q* ^+^ = 10 °C·min^–1^ could be evaluated, which significantly limited the sample set (a large number of the CHR measurements utilized *q* ^+^ = 30 °C·min^–1^ to enhance the calorimetric signal). The utilizable *C*_p_^max^-*q* ^–^ series were subject to an identical simulation-comparative procedure as described in Sections 3.1and 3.2. The results are listed in Table 3. As can be inferred from the Δ*x* and Δβ errors (difference between the true/published value and value estimated via the present simulation-comparative method), the majority of the predictions were within the ±0.05 confidence interval. The only major exception are only the *x* data for the (GeTe_4_)_40_(GaTe_3_)_60_ and (GeTe_4_)_50_(GaTe_3_)_50_ glasses. However, the corresponding literature source reports extremely high values of the nonlinearity parameter (*x* = 0.99), which may be an error itself, resulting, e.g., from an inaccurate curve-fitting procedure of instrumentation-distorted original DSC data. Nonetheless, considering that also the literature-reported data were determined with a certain level of uncertainty, and very often by a different evaluation methodology (usually curve-fitting or the less precise non-numerical advanced simulation-comparative method with much lower resolution in *x* and β), the performance of the present version of the simulation-comparative method is satisfactory. Note that the major weight of significance should still be given to the testing introduced in Sections 3.1and 3.2 due to the experimental inaccuracies/errors being nonexistent in the case of the simulated testing data series.

**Table 3:** Values of *x* and β TNM Parameters Reported in Ref 32 Are Denoted with the “publ” Index; *x* and β Values Estimated by the Present Simulation-Comparative Method from the Corresponding CHR Cycles Data Are Denoted with the “Pred” Index; the Differences between the Published and Predicted Values Were Calculated as *P*_publ_ – *P*_pred_ ^a^

composition	x_publ_	β_publ_	*x*_pred_	β_pred_	Δx	Δβ
As_2_Se_3_	0.57	0.71	0.54	0.74	0.03	–0.03
Se	0.42	0.58	0.44	0.62	–0.02	–0.04
Se_90_Te_10_	0.5	0.83	0.5	0.84	0	–0.01
Se_80_Te_20_	0.45	0.81	0.46	0.78	–0.01	0.03
Se_70_Te_30_	0.43	0.73	0.47	0.77	–0.04	–0.04
Ge_2_Se_98_	0.48	0.62	0.52	0.62	–0.04	0
Ge_4_Se_96_	0.43	0.48	0.46	0.52	–0.03	–0.04
Ge_6_Se_94_	0.43	0.43	0.37	0.4	0.06	0.03
Ge_8_Se_92_	0.3	0.4	0.32	0.4	–0.02	0
Ge_10_Se_90_	0.3	0.4	0.3	0.36	0	0.04
Ge_15_Se_85_	0.3	0.2	0.26	0.18	0.04	0.02
As_2_Se_98_	0.45	0.63	0.48	0.6	–0.03	0.03
As_4_Se_96_	0.45	0.63	0.44	0.62	0.01	0.01
As_6_Se_94_	0.47	0.65	0.43	0.61	0.04	0.04
As_8_Se_92_	0.43	0.58	0.42	0.58	0.01	0
As_10_Se_90_	0.4	0.6	0.38	0.6	0.02	0
As_15_Se_85_	0.48	0.57	0.48	0.56	0	0.01
GeSb_2_Se_4_	0.67	0.73	0.67	0.78	0	–0.05
Ge_2_Sb_2_Se_4.5_Te_0.5_ ^b^	0.58	0.62	0.58	0.6	0	0.02
Ge_2_Sb_2_Se_4_Te	0.58	0.56	0.58	0.56	0	0
Ge_20_Se_2_Te_78_	0.72	0.57	0.68	0.62	0.04	–0.05
Ge_20_Se_4_Te_76_	0.77	0.57	0.74	0.61	0.03	–0.04
Ge_20_Se_6_Te_74_	0.81	0.53	0.75	0.57	0.06	–0.04
Ge_20_Se_8_Te_72_	0.81	0.5	0.75	0.56	0.06	–0.06
(GeTe_4_)_40_(GaTe_3_)_60_	0.99	0.79	0.88	0.82	0.11	–0.03
(GeTe_4_)_50_(GaTe_3_)_50_	0.99	0.77	0.82	0.8	0.17	–0.03
(GeTe_4_)_60_(GaTe_3_)_40_	0.96	0.84	0.96	0.86	0	–0.02
(GeTe_4_)_67_(GaTe_3_)_33_	0.84	0.89	0.78	0.92	0.06	–0.03
(GeTe_4_)_75_(GaTe_3_)_25_	0.81	0.87	0.84	0.86	–0.03	0.01
(GeTe_4_)_86_(GaTe_3_)_14_	0.73	0.84	0.77	0.82	–0.04	0.02

a Note that the β value for Ge_15_Se_85_ reported in ref 32 was incorrect, and its correct value was taken from the original study.^57^

b These data were further used to demonstrate the robustness of the simulation-comparative method.

To demonstrate the advantages of the introduced simulation-comparative approach over the classic curve-fitting approach, two additional tests showing the computational efficiency and robustness of the simulation-comparative approach were performed. After uploading selected experimental data into the self-made software, the simulation-comparative database search and automatic determination of the best matching *x* and β values took approximately 1–2 s. On the other hand, the curve fitting of a single experimental curve takes approximately 30–300 s (depending on the required accuracy and nature of the data curve). Since at least 3–6 curves should be used to reliably determine the *x* and β values, the required time for the nonlinear optimization approach is ∼2–3 orders of magnitude higher compared to the simulation-comparative method. To demonstrate the robustness of the simulation-comparative approach, the data published in ref 53 for the Ge_2_Sb_2_Se_4.5_Te_0.5_ glass were used. Apart from the correctly formatted CHR cycle data (for which the results are presented in Table 3), the slightly incorrectly treated experimental data (with an inaccurately subtracted baseline) were also reported in ref^53^. The nonlinear optimization of these data resulted in major *q* ^–^-related trends in the data, estimating *x* between 0.40 and 0.65 and β between 0.50 and 0.65.^53^ On the other hand, the present simulation-comparative method provided the estimate of *x* ≈ 0.56 and β ≈ 0.58, which are indeed very close to the true values (*x* = 0.58 and β = 0.62; as reported in Table 3). This clearly demonstrates the robustness of the simulation-comparative approach over direct curve fitting when treating the unideal experimental data.

## 4 Conclusions

A robust and practically accessible implementation of the simulation-comparative method was introduced and systematically validated for application in the field of enthalpy relaxation of amorphous chalcogenide materials. By providing a library of precomputed *C*_p_^max^-*q* ^–^ data sets covering physically relevant ranges of enthalpy relaxation activation energies and glass transition temperatures for chalcogenide glasses, the present approach eliminates the need for direct TNM model simulations. This largely lowers the methodological and computational barriers that have so far limited the broader adoption of advanced structural relaxation analyses in amorphous material research. It was shown that the estimation of the TNM parameters *x* and β is, in the most common/relevant cases, done with a ±0.02 accuracy (97% of the cases). Even when extending to the more uncommon and rare combinations of the structural relaxation behavior (e.g., accommodating very high or very low *x* and β values, or by combining high Δh* with low *T*_p_ and vice versa), the prediction accuracy stays within the ±0.05 range. On average, the errors in estimating the parameter *x* were higher and more frequent compared to those associated with β estimation. Importantly, only a very weak dependence of the estimation accuracy on the discretization of the Δh* and *T*_p_ values in the core data sets was revealed, confirming the sufficient resolution of the data set matrix for reliable practical use. The comparison with experimental calorimetric data from the literature corroborated the applicability of the method to real materials, confirming the expected ±0.05 estimation accuracy for both TNM parameters (*x* and β). Since the introduced simulation-comparative framework offers a fast, transparent, and reliable route to TNM parameter determination using only standard CHR calorimetric measurements and basic data-processing tools, it is expected to facilitate wider and more consistent use of the enthalpy relaxation analyses across amorphous chalcogenide materials. Owing to the provided MATLAB code, the method is applicable with minimal adaptation applicable also to other classes of amorphous materials; the preparation and validation of additional core data sets suitable for polymeric materials will be reported in a sequel to the present paper.

## Supplementary Material







## Notes

MATLAB code for simulation of the TNM model (PDF)
Core data sets simulated for different combinations of TNM parameters­(ZIP)
Δ*h* ^***^ and *T*_p_ combinations used to test the simulation-comparative methodology (XLSX)
