# Supplementary material for: Simulation-Comparative Approach to Description of Glass Structural Relaxation Dynamics
Source: ACS Omega. 2026 Jul 30;11(31):47049–59. doi: 10.1021/acsomega.6c05942 (PMC13470727; doi:10.1021/acsomega.6c05942)
Supplement: Supplementary file 1 [file ao6c05942_si_001.pdf]

Electronic Supplementary Information File

Simulation-comparative approach to description of glass structural relaxation dynamics: The derivation for amorphous chalcogenide materials

Roman Svoboda

University of Pardubice, Faculty of Chemical Technology, Department of Physical Chemistry, Studentská 573, 532 10 Pardubice, Czech Republic.

Matlab code for simulating the TNM model data

……………………………………………………………………………………..

```
clear; clc;
A    = exp(-93);
dH   = 3e5;
x    = 0.6;
beta = 0.6;
Rg   = 8.314;
temp_program = [0   200;
             400   0;
             420   200];
time_min = temp_program(:,1);
T_points = temp_program(:,2);
dTstep = 0.1;
N_iso_points = 600;
T_full = [];
time_full = [];

for i = 1:length(T_points)-1
   T_start = T_points(i);
   T_end   = T_points(i+1);
   t_start = time_min(i) * 60;
   t_end   = time_min(i+1) * 60;

   if T_start == T_end
      t_rel = logspace(0,1,N_iso_points) - 1;
      t_rel = t_rel / max(t_rel);
      t_seg = t_start + t_rel * (t_end - t_start);
      T_seg = T_start * ones(1, N_iso_points);
   else
      N_ramp = max(round(abs(T_end - T_start)/dTstep),1);
      T_seg = linspace(T_start, T_end, N_ramp);
      t_seg = linspace(t_start, t_end, N_ramp);
   end

   T_full = [T_full, T_seg];
   time_full = [time_full, t_seg];
end

N = length(T_full);
T = T_full;
time_s = time_full;
T_K = T + 273.15;

dTvec = zeros(1,N); dTvec(2:end) = diff(T);
dtvec = zeros(1,N); dtvec(2:end) = diff(time_s);
q     = zeros(1,N); q(2:end) = dTvec(2:end)./dtvec(2:end);

for k = 2:N
   if abs(T(k) - T(k-1)) > 2*dTstep
```

```
        dTvec(k) = 0;
    end
end

Tf  = zeros(1,N);
tau = zeros(1,N);

Tf(1) = T(1);
tau(1) = A * exp( dH*( x*(1/Rg/T_K(1)) + (1-x)*(1/(Tf(1)+273.15)/Rg) ) );

for n = 1:N
    for k = 2:n
        tau(k) = A * exp( dH*( x*(1/Rg/T_K(k)) + ...
                      (1-x)*(1/(Tf(k-1)+273.15)/Rg) ) );
    end

    S = zeros(1,n);
    acc = 0;
    for k = n:-1:2
        if dTvec(k) ~= 0 && q(k) ~= 0
            dt_eff = abs(dTvec(k)/q(k));
        else
            dt_eff = dtvec(k);
        end
        acc = acc + dt_eff / tau(k);
        S(k) = acc;
    end

    if n > 1
        S(1) = max(S(2),0);
    else
        S(1) = 0;
    end

    sum_term = 0;
    for j = 1:n
        if dTvec(j) ~= 0
            sum_term = sum_term + dTvec(j)*(1 - exp(-(S(j))^beta));
        end
    end

    Tf(n) = T(1) + sum_term;
end
```
…………………………………………………………………………………………..
